# Sunscreen Products as Emerging Pollutants to Coastal Waters

**DOI:** 10.1371/journal.pone.0065451

**Published:** 2013-06-05

**Authors:** Antonio Tovar-Sánchez, David Sánchez-Quiles, Gotzon Basterretxea, Juan L. Benedé, Alberto Chisvert, Amparo Salvador, Ignacio Moreno-Garrido, Julián Blasco

**Affiliations:** 1 Department of Global Change Research, Mediterranean Institute for Advanced Studies (UIB-CSIC), Esporles, Balearic Island, Spain; 2 Department of Ecology and Marine Resources, Mediterranean Institute for Advanced Studies (UIB-CSIC), Esporles, Balearic Island, Spain; 3 Department of Analytical Chemistry, Facultad de Química, Universitat de València, Burjassot, Valencia, Spain; 4 ICMAN-Instituto de Ciencias Marinas de Andalucía (CSIC), Puerto Real, Cádiz, Spain; University of California, Merced, United States of America

## Abstract

A growing awareness of the risks associated with skin exposure to ultraviolet (UV) radiation over the past decades has led to increased use of sunscreen cosmetic products leading the introduction of new chemical compounds in the marine environment. Although coastal tourism and recreation are the largest and most rapidly growing activities in the world, the evaluation of sunscreen as source of chemicals to the coastal marine system has not been addressed. Concentrations of chemical UV filters included in the formulation of sunscreens, such as benzophehone 3 (BZ-3), 4-methylbenzylidene camphor (4-MBC), TiO_2_ and ZnO, are detected in nearshore waters with variable concentrations along the day and mainly concentrated in the surface microlayer (i.e. 53.6–577.5 ng L^-1^ BZ-3; 51.4–113.4 ng L^-1^ 4-MBC; 6.9–37.6 µg L^-1^ Ti; 1.0–3.3 µg L^-1^ Zn). The presence of these compounds in seawater suggests relevant effects on phytoplankton. Indeed, we provide evidences of the negative effect of sunblocks on the growth of the commonly found marine diatom *Chaetoceros gracilis* (mean EC_50_ = 125±71 mg L^-1^). Dissolution of sunscreens in seawater also releases inorganic nutrients (N, P and Si forms) that can fuel algal growth. In particular, PO_4_^3−^ is released by these products in notable amounts (up to 17 µmol PO_4_^3−^ g^−1^). We conservatively estimate an increase of up to 100% background PO_4_^3−^ concentrations (0.12 µmol L^-1^ over a background level of 0.06 µmol L^-1^) in nearshore waters during low water renewal conditions in a populated beach in Majorca island. Our results show that sunscreen products are a significant source of organic and inorganic chemicals that reach the sea with potential ecological consequences on the coastal marine ecosystem.

## Introduction

In spite of the fact that coastal tourism and recreation are becoming the largest and most rapidly growing activities in the world [1] and that sunscreen products have been used for nearly 80 years, the effect of sunscreens, as a source of introduced chemicals to the coastal marine system, has not yet been addressed. Sun protection cosmetics are composed of organic (para-aminobenzoates, cinnamates, benzophenones, dibenzoylmethanes, camphor derivatives and benzimidazoles, which absorb the UV radiations), and/or inorganic UV chemical filters (i.e. TiO_2_ and ZnO) that reflect and scatter the UV radiation protecting human skin from direct radiation of sunlight [2], [3]. There are around 45 UV chemical filters subjected to regulation in different countries [3], [4]. In addition to these UV filters, sunscreen products contain other ingredients such as preservatives (e.g. parabens derivates) [5], coloring agents (e.g. ammonium sulphate, copper powder, ferric ammonium ferrocyanide, iron and zinc oxides, etc.) [6], film forming agents (e.g. acrylates and acrylamides) [7], surfactants, chelators, viscosity controllers (e.g. potassium cetyl phosphate, pentasodium ethylenediamine tetramethylene phosphonate among others) [8] and fragrances, etc. Formulation and concentration of cosmetic ingredients in commercial sunscreens are varied, and legislated by local or international agencies (e.g. European Union Cosmetics Directive [9] or United States Food and Drug Administration [10]) to reach a compromise between adequate UV protection and minimal side effects for humans [2]. Studies conducted in lakes (i.e. Zurich and Hüttnersee Lakes, Swiss) suggest that UV filter removal processes from the water column are important, and can be mediated by biodegradation processes and/or absorption sedimentation [11]. Because of their lipophilicity, persistence and stability against biodegradation they have been shown to accumulate in the food chain [4], [12].

Coastal tourism is considered one of the fastest growing forms of tourism in recent decades [1] being the Mediterranean one of the most important tourism regions in the world [13]. For decades, the Balearic Islands (Western Mediterranean Sea) have provided the traditional sun, sand and sea product. Tourism is the first economic activity in the Islands. The islands comprise a total surface area of 5040 km^2^, 1428 km of coastline and have usually been considered in the literature as a typical example of a second-generation european mass tourist resort [14]. Majorca island (the largest of the Balearic archipelago), with about one million inhabitants, received 9.8 million international arrivals in 2010 [15], presenting one of the highest tourist rates per capita in the world [16].

In this study we estimate the potential effect of commercial sunscreen released in nearshore waters by beachgoers. We conduct field and laboratories studies to evaluate the presence of chemicals products released from sunscreens in coastal seawater and its effect on the marine phytoplankton. Particularly, (1) we present the results for UV chemical filters levels in different fractions of surface marine waters of three Majorca areas; (2) we evaluate the contribution of sunscreen products to the total dissolved P in nearshore waters of a populated beach in Majorca island; and (3) we test the effect of sunscreens on the growth rate of a marine diatom (i.e. *Chaetoceros gracilis*).

## Materials and Methods

### Ethics Statement

A permit for sampling in the bathing areas of Palmira and Santa Ponça beaches were obtained from the city hall of Calviá (Majorca Island). No specific permit was required for sampling in the Ses Salines Cape. The maritime area of Ses Salines is not private and protected for sampling. The study did not involve endangered or protected species.

### Field Sampling

**Figure 1:**
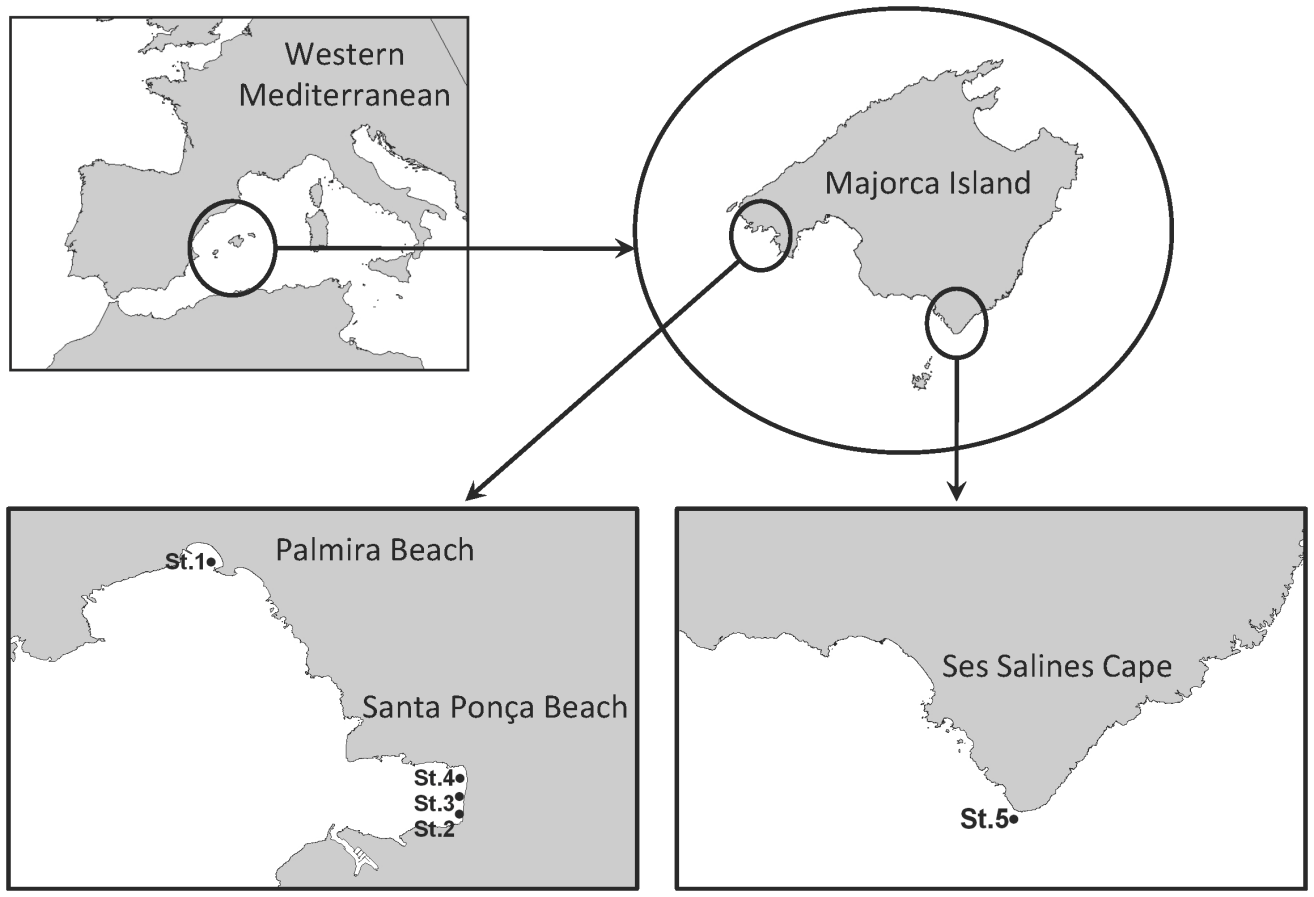
Sampling locations: St.1 (Palmira Beach); St.2, St.3, St.4 (Santa Ponça beach); St.5 (Ses Salines Cape).

Surface nearshore waters of three beaches around Majorca Island were sampled in August-September 2011. Two areas corresponded to semi-enclosed and densely populated beaches in resort areas (maximum daily density of 3.5–4.5 users m^-1^ shoreline), and the third one, considered a control, was an open and scarcely used beach located in a pristine area (Figure 1).

### Seawater Collection

Surface waters (microlayer and subsurface) were collected from a zodiac during August 20^th^, 21^st^ in the swimming area of Palmira and Santa Ponça beaches, and in September 1^st^ at Ses Salines Cape, respectively. Surface seawater (1 m depth) was collected using a peristaltic pump and pumped through acid-cleaned Teflon tubing coupled to a C-flex tubing (for the Cole-Parmer peristaltic pump head), filtered through an acid-cleaned polypropylene cartridge filter (0.22 µm; MSI, Calyx®) for the dissolved fraction, and collected in a 0.5 L low-density polyethylene plastic bottle [17]. Surface microlayer (SML) samples were collected using a glass plate sampler [18].

### Chemical Analysis

Organic UV filters (i.e. BZ-3 and 4-MBC) in seawater were preconcentrated by dispersive liquid-liquid microextraction (DLLME). Stock solutions (500 ng mL^-1^) of BZ-3 and MBC in ethanol were prepared. Then, a multicomponent aqueous stock solution (25 ng mL^-1^) of BZ-3 and MBC was prepared from these, and it was used to prepare multicomponent aqueous working solutions (50–250 ng L^−1^ or 200–1000 ng L^-1^). On the other hand, a stock solution (20 µg mL^-1^) of deuterated benzophenone (benzophenone-d10 (BZ-d10)), which was used as surrogate, was prepared. Then, an aqueous working solution (65 ng mL^-1^) was prepared from this.

Ten mL of each of the standard working solutions were pH adjusted with glacial acetic acid to 2–4, then 1–1.5 g of sodium chloride were added to reach a final content within 10–15% (m/v), and finally 25 µL of the working surrogate solution were added. Then, they were subjected to DLLME in 15-mL polyethylene centrifuge tubes, by rapidly injecting pre-mixed 940 µL of acetone with 60 µL of chloroform solutions. Once the cloudy solutions were formed, they were centrifuged at 6000 rpm for 5 min. After centrifugation, approximately 25 µL of each of the organic sedimented phases were collected with the aid of a 50 µL syringe and transferred into 100 µL inserts placed inside 1.5 mL injection vials. Then, 2 µL were injected into the GC system coupled to a mass spectrometry (MS) detector operated in positive electron ionisation mode at ionisation energy of 70 eV and with a multiplier voltage set at 1400 V. The inlet temperature was 280 °C and the injection was accomplished in splitless mode (splitless time: 1 min). The separation was run at a 1 mL min^−1^ helium constant flow rate. The oven temperature program was: from 70 °C (1 min) to 170 °C at 10 °C min^−1^, then to 200 °C at 2 °C min^−1^ and finally to 280 °C (6 min) at 10 °C min^−1^. The transfer line and ion source temperatures were set at 280 and 250 °C, respectively. The chromatograms were recorded in selected ion monitoring (SIM) mode at the following mass/charge (m/z) ratios: 151, 227 (quantifier) and 228 for BZ3; 128, 211 and 254 (quantifier) for 4-MBC; and 82, 110 (quantifier) and 192 for BZ-d10.

Calibration was performed by plotting A_i_/A_sur_ (where A_i_ is the peak area of the target analyte and A_sur_ that of the surrogate (i.e., BZ-d10), each one obtained by using its quantifier ion) versus target analyte concentration.

For the determination of the soluble fraction of organic UV filters, 10 mL of filtered water samples were pH adjusted, and sodium chloride and surrogate solution added as previously described. For the determination of the total content (i.e., soluble plus particulate fraction), 10 mL of unfiltered water samples were sonicated for 10 min, then they were filtered and treated as described before for the determination of the soluble fraction. Then, they were subjected to DLLME and injected into GC-MS system as previously described for standards. For each target analyte, Ai/Asur was obtained and interpolated in the corresponding calibration line, and the concentration was finally obtained.

Titanium in seawater samples was analyzed with MSFIA-LWCC [19], after prior digestion with potassium peroxodisulfate. Recovery of spikes of Ti in seawater was 101.5±8.8%. Inorganic nutrients in seawater (i.e. PO_4_^3−^, NO_3_^−^, NO_2_^−^, SiO_2_ and NH_4_^+^) were determined with an autoanalyzer (Alliance Futura) using colorimetric techniques [20]. The accuracy of the analysis was established using Coastal Seawater Reference Material for Nutrients (MOOS-1, NRC-CNRC), with recoveries of 100.7%, 100.5%, 97.4% and 86.8% for PO_4_^3−^, NO_3_^−^, NO_2_^−^ and SiO_2_, respectively. Zinc in seawater was preconcentrated by the APDC/DDDC organic extraction method [17] and analyzed by ICP-MS (PerkinElmer ELAN DRC-e). The accuracy of the analysis in seawater was established using Coastal water Reference Material for Trace Metals (CASS-4, NRC-CNRC) with recoveries of 95.6%. Metals in sunscreen were analyzed by ICP-AES (Perkin Elmer ICP-AES Optima 5300 DV) after previous chemical digestion [19]. Reference materials of sunscreen consist in three sunscreens with three different sun protection factors (SPF) made in our laboratory, with known concentrations for Ti and Zn. Recoveries were 108±6% for Ti and 101±1% for Zn. All sampling and analytical operations were performed following trace-metal clean techniques. All chemical analysis in samples and stocks solutions were analyzed by triplicate.

### Nutrient Release Experiment

The experiment on the release of nutrients from sunscreen was carried out by dissolving 15 g of the sunscreen number 11 (Table S1) into 500 mL of artificial seawater (37 g of NaCl per L), and stirred up to five days in an orbital shaker (300 rpm) inside a culture chamber at 25°C. Dissolved phosphate estimations in nearshore waters at Palmira beach (Figure S1) are based on the release kinetics experiment. The number of beachgoers was simulated from maximum midday direct counts and allowing for a sinusoidal variation during day length (13 h; Figure S2). Each hour, 25% of the beachgoers were assumed to swim, and only 10% of the sunscreen was dissolved in seawater. A median content of 0.08 µmolP g^-1^ sunscreen was used. Water renewal at the beach was calculated from a series of 6 hours averages of historical current measurements in the sampling site during the same season, obtained using moored Nortek ADCPs [21].

### Microalgal Toxicity Biossays

Standard microalgal toxicity bioassays were carried out on ASTM [22] Substitute Ocean Water enriched with f/2 medium on 10^4^ initial cellular density populations of Chaetoceros gracilis, obtained from the ICMAN marine culture collection, included in the BIOCISE index. Exposition was carried out at 20±1°C under continuous white light (35.2±1.1 µmol(quantum) m^−2^ s^−1^) in a controlled culture chamber (Ibercex) in 50 mL of exposition media disposed in 125 mL borosilicate conical flasks topped with synthetic cotton -Perlon-. After a previous wide-range concentration experiment developed in order to get a narrower concentration interval [23], five concentrations plus a control were disposed by triplicate for each compose tested. After 72 hours exposition, cellular counts were performed under light microscopy on Neubauer counting chambers. Considering controls as 100% growth, percentage of growth inhibition was calculated for each cream concentration. Adjusting values of growth inhibition following Hampel et al., 2001 [24], EC50% 72h, it means, effective concentration of pollutant which inhibits microalgal population growth (biomass) at 72 hours [25] was calculated for each cream on this microalgal species.

## Results and Discussion

**Figure 2:**
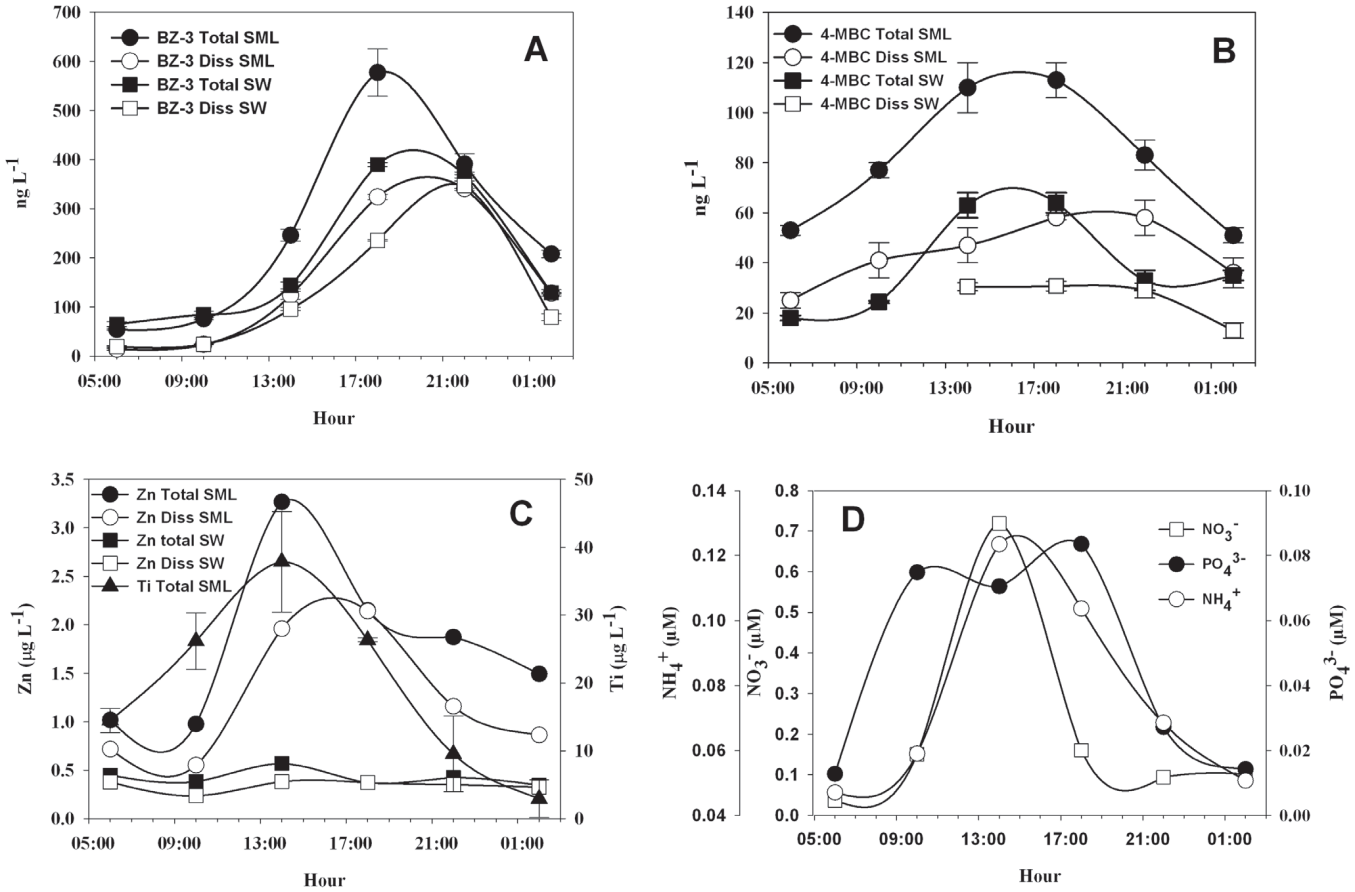
Concentration of BZ-3 (A), 4-MBC (B), Zn (C), Ti (C) and nutrients (D) in the unfiltered (Total) and filtered (<0.22 µm; Diss) fraction of the Surface microlayer (SML) and Subsurface (1 cm) seawater (SW) samples from Palmira Beach. Nutrients concentrations are only plotted in the dissolved fraction of the SML (D). Error bars represent the standard deviation (n = 3).

Chemical analysis of the surface nearshore waters of three areas around Majorca Island showed that four of the main chemicals used in commercial sunscreens were detected in the surface waters, with the highest concentrations measured in the unfiltered fraction of the surface microlayer (SML) (i.e. BZ-3∶580±50 ng L^-1^; 4-MBC: 113±7 ng L^-1^; and Ti: 38±7 µg L^-1^; Zn: 10.8 µg L^-1^; Table 1). Because of the lipophilic characteristic of these cosmetics [4], and the insolubility of many of their chemicals, sunscreen products tend to be more concentrated in the surface microlayer (SML) and to accumulate in soils and particles [26]. Levels of these chemicals co-varied throughout the day reaching the highest concentrations between 14∶00 and 18∶00 h (Figure 2A–D), a few hours after the beachgoers maximum numbers occurring around noon, and when sunlight radiation is maximum and sunscreen application is expected to be at the highest level of application [4]. In the case of the unfiltered fraction of the four compounds (i.e. BZ3, MBC, Zn and Ti) in the surface microlayer, midday concentrations exceeded between 60 and 90% background values (observed during night or early morning), suggesting a common source for these products. Even in the pristine beach of Ses Salines Cape (Figure 1) detectable concentrations of BZ-3 and 4-MBC (16±3 and 26±1 ng L^-1^, respectively) were measured in the total fraction of SML, and in subsurface water (36±2 and 27±2 ng L^-1^, respectively), whilst Ti was only detected in the total water fraction of the SML (23.7±1.7 ng L^-1^). This suggests a high degree of alongshore connectivity due to the persistence of these products that results in their generalized impact around the island.

**Table 1:** Midday concentration ± standard deviation (n = 3) of BZ-3 (ng L^-1^), 4-MBC (ng L^-1^), Ti (µg L^-1^), Zn (µg L^-1^) and nutrients (nmol.L^-1^) in the unfiltered (Total) and filtered (<0.22 µm; Diss) fraction of the Surface microlayer (SML) and Subsurface (1 cm) seawater (SW) samples.

Beach	Sampling stations	Total SML				Diss SML								
		BZ-3	4-MBC	Ti	Zn	BZ-3	4-MBC	Ti	Zn	PO_4_^3−^	NO_3_^−^	NO_2_^−^	SiO_2_	NH_4_^+^
**Palmira**	**St.1**	245.6±12.0	109.6±10.0	37.6±7.3	3.3	123±8.4	46.7±6.7	nd	2.0	70.5	718.8	37.8	5091.9	123.4
**Santa Ponça**	**St.2**	-	-	-	-	-	-	-	-	-	-	-	-	-
**Santa Ponça**	**St.3**	174.8±10.5	59.8±3.9	12.1±1.2	10.8	156.1±6.0	55.4±1.2	nd	7.7	8.5	452.5	3.9	5032.8	55.3
**Santa Ponça**	**St.4**	-	-	-	-	-	-	-	-	-	-	-	-	-
**Ses Salines (control)**	**St.5**	15.8±3.0	25.7±1.2	23.7±1.7	0.8	nd	nd	nd	0.5	153.5	933.0	38.5	969.6	66.5

		Total SW				Diss SW								
		BZ-3	4-MBC	Ti	Zn	BZ-3	4-MBC	Ti	Zn	PO_4_^3−^	NO_3_^−^	NO_2_^−^	SiO_2_	NH_4_^+^
**Palmira**	**St.1**	143.6±7.4	62.5±4.5	nd	0.6	95.8±2.9	30.5±1.6	nd	0.4	14.2	161.2	nd	6653.5	129.5
**Santa Ponça**	**St.2**	76.2±4.6	65.0±5.0	nd	1.0	40.4±2.6	37.4±3.1	nd	0.9	nd	1218.1	nd	6209.9	81.9
**Santa Ponça**	**St.3**	155.4±5.6	51.4±2.5	nd	0.7	119.6±1.7	29.3±5.0	nd	0.7	nd	15.6	nd	5414.3	84.4
**Santa Ponça**	**St.4**	314.8±2.9	47.5±3.9	nd	0.7	241.9±5.2	14.7±4.0	nd	0.9	nd	299.0	100.3	6541.2	81.5
**Ses Salines (control)**	**St.5**	36.3±2.2	26.6 ± 2.0	nd	0.1	nd	nd	nd	0.1	95.1	2892.3	7.0	702.1	66.2

(-) not collected; nd: not detected.

**Figure 3:**
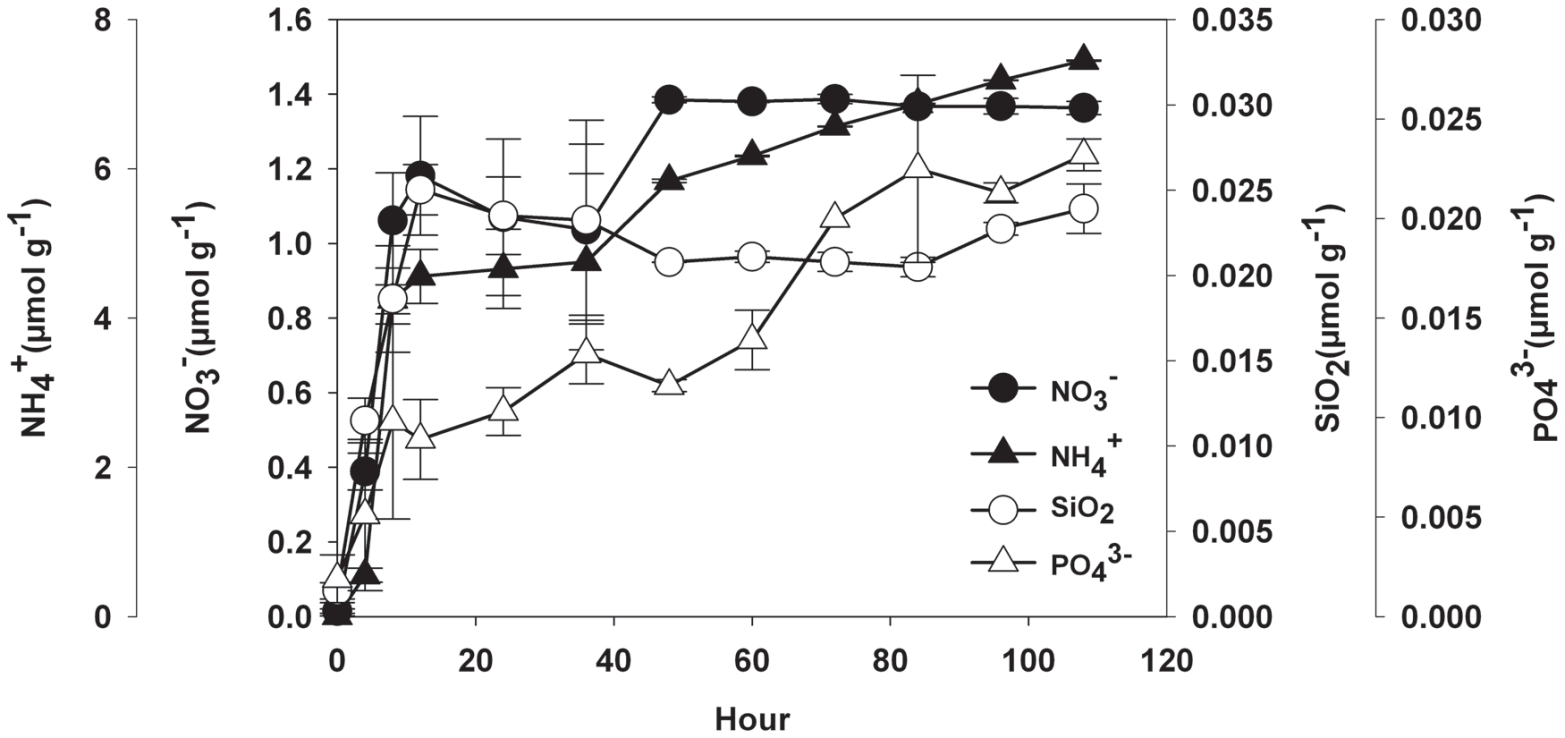
Kinetics of nutrient release from a commercial sunscreen in seawater (n = 3). NO_2_^-^ was not detected.

Our study also shows the release of some inorganic nutrients (i.e. PO_4_^3-^, NO_3_^-^, and NH_4_^+^) which may affect algal growth. A release kinetic experiment consisting of shaking a commercial sunscreen (num. 11 in Table S1) in artificial seawater (37g L^-1^ NaCl) showed rapid dissolution of silicate and nitrogen compounds (in the first 16 hours), and lower rates (if any) thereafter (Figure 3). Conversely, the release dynamics of PO_4_^3-^ was fairly more progressive and linear (0.01 µmol g^-1^ h^-1^). Nutrient release kinetics varies depending on sunscreen composition, but since a majority of the tested products stabilized their release after 72 h we assumed that concentrations at this time to be indicative values for total content. The 13 commercial sunscreens tested provided final concentrations in water of 2±5 µmol g^-1^ of PO_4_^3-^, 0.2±0.4 µmol g^-1^ of NO_3_^-^, 0.001±0.002 µmol g^-1^ of NO_2_^-^, 2±2 µmol g^-1^ of SiO_2_ and 0.02±0.01 µmol g^-1^ of NH_4_^+^ (Table S1). It is particularly notable that on the average the release of PO_4_^3-^ occurs in relatively high molar ratios compared to nitrogen forms. This high PO_4_^3-^ mean concentration is nevertheless based on only a few products containing high phosphate concentrations (up to 17 µmol g^-1^; Table S1), but its importance should not be ignored because they are widely consumed.

Inorganic nutrient inputs stimulate primary production in oligotrophic waters, as in the western Mediterranean Sea [27], so that recreational activities at some sites may represent a significant and previously overlooked nutrient source for the nearshore environment. Based on the estimated sunscreen dose (half of the recommended 2 mg cm^-2^ and ∼36 g/adult person) [28] direct counts on the number of beachgoers, and water renewal estimations depicted from wind conditions, we conservatively estimated that on a calm day (low water renewal) sunscreens may increase by an average of 55% above the otherwise low PO_4_^3-^ concentrations in Palmira Beach (Figure S1). Under low water renewal conditions, up to 0.12 µmol L^-1^, this represents an approximate doubling of mean offshore concentrations. While other land sources are considered quantitatively more important, this modest, but significant contribution in a P-limited area could play an important role in the dynamics of nearshore phytoplankton. It has been demonstrated that concentrations of 20 nmol L^-1^ of P induce significant phytoplankton response in the coastal Mediterranean sea during summer [27]. Furthermore, in addition to inorganic nutrients and chemical UV filters, sunscreens contain other constituents such as Al and Fe. These metals (together with Ti and Zn) were detected in at least one of the 13 commercial sunscreens analyzed (five of which are among the ten bestsellers in Spanish pharmacies in 2011, according to Sell Out database from IMS Health® [29]) (Table S2). Iron, together with P, is an essential micronutrient for phytoplankton growth and it is also suggested to limit primary production in the western Mediterranean [30].

**Figure 4:**
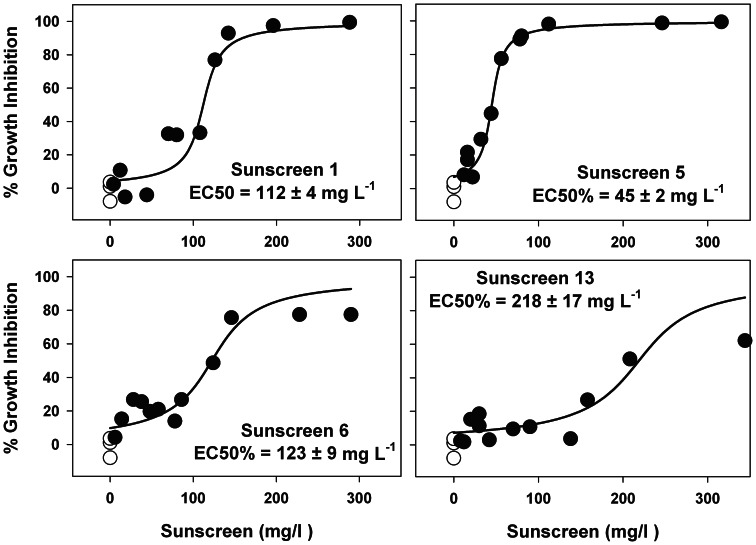
Growth inhibition rate for *Chaetoceros gracilis* exposed to different concentrations of commercial sunscreens after 72 hours culture. White circles represent controls.

We tested the effect of sunscreens on the growth rate of the marine phytoplankton *Chaetoceros gracilis*, which is a widespread species in the western Mediterranean [31]. The acute toxicity was measured by calculating half maximal effective concentration (EC_50_) after 72h incubation, resulting in an average of 125±71 mg L^-1^. The sunscreen num. 5 (a solar spray; Table S1) induced the highest level of toxicity with a EC_50_ of 45±2 mg L^-1^, while sunscreen num. 13 (a solar milk) presented the lowest effects (EC_50_ = 218±17 mg L^-1^) (Figure 4). The concentrations of sunscreens at which EC50 occurs (45–218 mg L^-1^) are higher than environmental concentrations measured in our studied areas. These amounts of sunscreen (at which EC50 occurs) are reflecting the threshold for acute toxicity. However, even at very low sunscreen concentrations certain inhibitory effect is observed (Figure 4). The higher toxicity of the spray versus cream or milk formats could be due to its higher content of hydrosoluble compounds, making them more bio-available to phytoplankton. Our results demonstrate the toxicity of the commercial sunscreen for marine phytoplankton, and confirm previous studies of toxicity carried out with individual organic and inorganic UV filters on marine organisms (including green algae, crustacean, phytoplankton and fishes) [32]–[38].

### Conclusions

More than half of today’s world population live in coastal areas, and estimates for the future suggest that in three decades from now nearly 75 percent of the world’s population will live along coasts [39]. This fact, combined with data showing that sun protection products are one of the fastest growing products globally [40], points to sunscreens as a potential pollutant with implications for the coastal marine ecosystem. The results presented here suggest that sunscreens in coastal waters may produce deleterious effects in the coastal ecosystem, either, by inhibiting growth of some marine phytoplankton species or by adding essential micronutrients which may stimulate the growth of others.

## Supporting Information

Figure S1 **Modeled variations of PO_4_^3−^ (µM) at Palmira Beach (Majorca) estimated from sinusoidal variations of beachgoers and 6 hours averaged current velocities.** Dashed line indicates shelf water background concentration.
(TIF)

Figure S2 **Diel variation of beach users at Palmira during a labor day (bars) and sinusoidal adjustment used in model simulations (line).**
(TIF)

Table S1 **Concentration (average ± SDV) of nutrients in nmol g^-1^ released from commercial sunscreens in seawater after 72 h shaking.** Samples were analyzed by triplicate except for nutrients from sunscreen 4. SPF (Sun Protection Factor).
(DOCX)

Table S2 **Concentration of metals in µg g^-1^ (average ± SDV, n = 3) in commercial sunscreens.** SPF (Sun Protection Factor). Other elements such as Ag, Cd, Co, Cr, Cu, Mn, Mo, Ni, Si, Sr, Pb, Tl, V, and Zr were not detected.
(DOCX)
