# Supplementary figures and images for: Sunscreen Products as Emerging Pollutants to Coastal Waters
Source: PLoS One. 2013 Jun 5;8(6):e65451. doi: 10.1371/journal.pone.0065451 (PMC3673939; doi:10.1371/journal.pone.0065451)

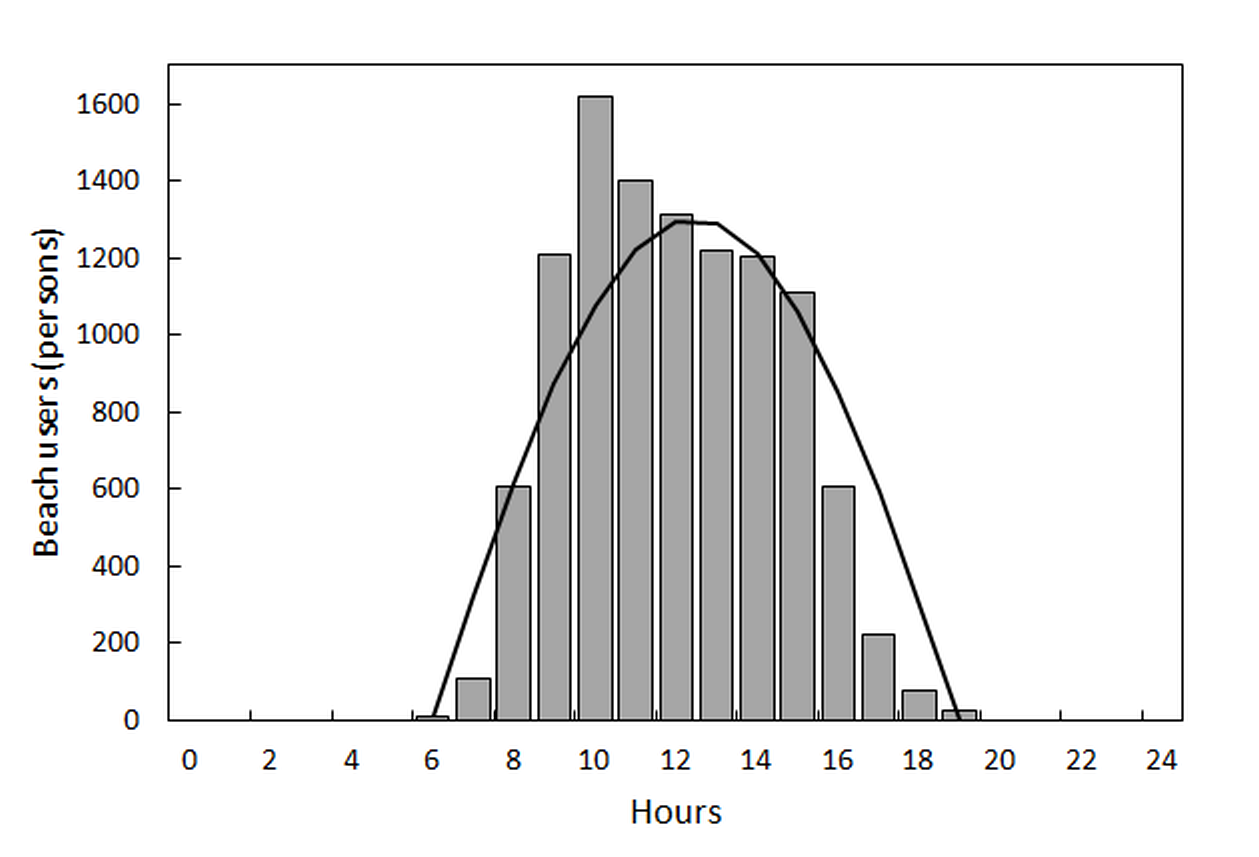

Supplement: Figure S2 — Diel variation of beach users at Palmira during a labor day (bars) and sinusoidal adjustment used in model simulations (line). (TIF) [file pone.0065451.s002.tif]
