# Supplementary material for: Sunscreen Products as Emerging Pollutants to Coastal Waters
Source: PLoS One. 2013 Jun 5;8(6):e65451. doi: 10.1371/journal.pone.0065451 (PMC3673939; doi:10.1371/journal.pone.0065451)
Supplement: Table S1 — Concentration (average ± SDV) of nutrients in nmol g-1 released from commercial sunscreens in seawater after 72 h shaking. Samples were analyzed by triplicate except for nutrients from sunscreen 4. SPF (Sun Protection Factor). (DOCX) [file pone.0065451.s003.docx]

**Table S1**. Concentration (average ± SDV) of nutrients in nmol g^-1^ released from commercial sunscreens in seawater after 72 h shaking. Samples were analyzed by triplicate except for nutrients from sunscreen 4. SPF (Sun Protection Factor).
