# Supplementary material for: Sunscreen Products as Emerging Pollutants to Coastal Waters
Source: PLoS One. 2013 Jun 5;8(6):e65451. doi: 10.1371/journal.pone.0065451 (PMC3673939; doi:10.1371/journal.pone.0065451)
Supplement: Table S2 — Concentration of metals in µg g-1 (average ± SDV, n = 3) in commercial sunscreens. SPF (Sun Protection Factor). Other elements such as Ag, Cd, Co, Cr, Cu, Mn, Mo, Ni, Si, Sr, Pb, Tl, V, and Zr were not detected. (DOCX) [file pone.0065451.s004.docx]

**Table S2**. Concentration of metals in µg g^-1^ (average ± SDV, n=3) in commercial sunscreens. SPF (Sun Protection Factor). Other elements such as Ag, Cd, Co, Cr, Cu, Mn, Mo, Ni, Si, Sr, Pb, Tl, V, and Zr were not detected.
